# Efficacy and safety of Shenmai injection and Shenfu injection on postoperative cognitive dysfunction: a systematic review and meta-analysis

**DOI:** 10.3389/fphar.2026.1664949

**Published:** 2026-06-30

**Authors:** Qianqian Liu, Lina Cheng, Yongchao Yin, Jiafu Ma, Wenjian Pei, Yahui Pan

**Affiliations:** 1 Department of Chinese and Western Medicine, Shandong Provincial Hospital Affiliated to Shandong First Medical University, Jinan, China; 2 Department of Encephalopathy, Hebei Provincial Hospital of Traditional Chinese Medicine, Shijiazhuang, China; 3 Department of Scientific Research, Shandong Provincial Hospital Affiliated to Shandong First Medical University, Jinan, China; 4 Emergency Department, People’s Hospital Affiliated to Shandong First Medical University, Jinan, China; 5 Department of Rehabilitation Medicine, Shandong Provincial Hospital Affiliated to Shandong First Medical University, Jinan, China

**Keywords:** meta-analysis, mini-mental state examination, postoperative cognitive dysfunction, Shenfu injection, Shenmai injection, traditional Chinese medicine

## Abstract

**Background:**

Postoperative cognitive dysfunction (POCD) is a common neurological complication after surgery, associated with increased adverse events and mortality. While studies suggest that Shenmai injection (SMI) or Shenfu injection (SFI) may be associated with reduce POCD, rigorous systematic reviews specifically on these two botanical drug injections are lacking.

**Methods:**

We comprehensively searched eight databases (PubMed, Embase, Cochrane Library, Web of Science, CNKI, Wanfang, VIP, CBM) from inception to May 2025 for RCTs on SMI and SFI for POCD. Data were analyzed using RevMan 5.4. Primary outcomes were Mini-Mental State Examination (MMSE) score and POCD incidence. Secondary outcomes included serum S100β protein concentration and postoperative consciousness recovery time. Continuous outcomes used mean difference (MD) or standardized mean difference (SMD) with 95% CI; dichotomous outcomes used relative risk (RR) with 95% CI. This study was registered in INPLASY (registration number: 202470131).

**Results:**

A total of 552 records were identified, and after deduplication and screening, sixteen RCTs (N = 1,199 patients) were finally included. Both SMI and SFI were associated with statistically significant higher postoperative MMSE scores at multiple time points (all P < 0.01) and were associated with lower POCD incidence at 3 days (SMI RR 0.38; SFI RR 0.52) and 7 days (SMI RR 0.26; SFI RR 0.47). However, all included trials were at high risk of bias (lack of allocation concealment and blinding). SFI was associated with lower serum S100β at 24 h (SMD = −0.40); the effect of SMI could not be evaluated due to missing data. Both injections were associated with shorter consciousness recovery time (MD = −5.67 min, 95% CI: −6.46 to −4.87, P < 0.001). Subgroup analyses showed a trend toward reduced POCD incidence across doses. Most studies did not report detailed preparation composition or chemical characterization. Only three of the 16 included RCTs reported adverse events, and therefore no reliable safety conclusions can be drawn from the current RCT evidence. Sensitivity analysis confirmed robustness. GRADE evidence quality was low for all outcomes.

**Conclusion:**

Low-certainty evidence suggests a possible association of SMI or SFI with improved cognitive outcomes, but causality is unproven. These preliminary findings require confirmation in rigorous RCTs with proper blinding and standardized measures.

**Systematic Review Registration:**

https://inplasy.com/inplasy-2024-7-0131, identifier 202470131.

## 1 Introduction

Postoperative cognitive dysfunction (POCD), a common complication of anesthesia and surgery (Evered et al., 2018; Kitsis et al., 2022), manifests as cognitive decline including impaired memory, diminished attention, and deficits in other cognitive domains, potentially lasting weeks to years (Inouye et al., 2014; Inouye et al., 2016). It increases postoperative complications, prolongs hospital stays (Berger et al., 2018; Kitsis et al., 2022), reduces quality of life, and raises mortality (O’Brien et al., 2017; Rudolph and Marcantonio, 2011). Data indicate POCD incidence ranges from 9% to 54% in cardiac surgery patients, with rates of 26% at 1 week and 10% at 3 months postoperatively in patients over 60 (Bhushan et al., 2021). Furthermore, early postoperative cognitive impairment is associated with long-term cognitive deficits and may represent a potential risk factor for dementia (Relander et al., 2020; Kahl et al., 2021; Trubnikova et al., 2017; Florido-Santiago et al., 2023). Therefore, timely preventive interventions could enhance cognitive reserve, facilitate recovery from neurological impairment, reduce long-term surgery-related cognitive decline, and ultimately improve overall quality of life and wellbeing (O' Brien et al., 2017; Liu et al., 2022; Kunicki et al., 2023).

The mechanisms underlying POCD remain unclear, and no unified strategies for its prevention or treatment exist. While numerous studies worldwide have explored various approaches—such as anesthetic selection, anesthetic techniques and depth (Zeng et al., 2023; Viderman et al., 2023; Tu et al., 2022), perioperative pain management (Khaled et al., 2022), perioperative cognitive training (Zhao et al., 2023), and identification of high-risk patients with modifiable risk factors (Evered et al., 2022)—their effectiveness remains debated (O' Brien et al., 2017; Liu et al., 2022). In recent years, complementary and alternative medicine (CAM) has gained considerable attention for the potential prevention and treatment of POCD (Zhang et al., 2018; Wang et al., 2023; Guo et al., 2022; Peng et al., 2020; Chen et al., 2018). It is important to note that *in vitro* and animal studies can generate hypotheses and provide pharmacological plausibility, but they do not constitute clinical evidence. This systematic review focuses solely on human randomized controlled trials.

Shenmai Injection (SMI) and Shenfu Injection (SFI), botanical drug injections derived from traditional Chinese medicine, approved by China’s National Medical Products Administration (NMPA) and designated as national protected varieties, are now widely used for POCD prevention (Chen et al., 2018; Zhou et al., 2023; Yang et al., 2017). Experimental studies indicate SMI inhibits oxidative stress and autophagy-related protein expression (Wu et al., 2022; Yang et al., 2016), while SFI improves glucose uptake, mitochondrial function (Zhang et al., 2014), cerebral microcirculation, and reduces inflammatory injury (Wu et al., 2021). Observational studies suggest both possess advantages and potential in improving neurological deficits and promoting neural repair/regeneration. However, reliable clinical evidence for POCD prevention remains lacking. Therefore, we conducted a meta-analysis to evaluate the efficacy and safety of SMI and SFI for POCD prevention. The overall objective of this systematic review is to systematically evaluate the efficacy and safety of SMI and SFI for the prevention of POCD. The specific objectives are to: (1) compare the effects of SMI or SFI versus controls on postoperative MMSE scores; (2) assess the difference in POCD incidence between the two groups; (3) analyze postoperative serum S100β protein concentration and time to recovery of consciousness; (4) summarize the occurrence of adverse events; and (5) assess the risk of bias of the included studies using the Cochrane Risk of Bias tool for Randomized Trials (RoB 2.0, 2019 version) and systematically evaluate their methodological quality (Sterne et al., 2019). Primary outcomes were postoperative Mini-Mental State Examination (MMSE) scores and POCD incidence; secondary outcomes included postoperative serum S100β protein concentration, time to recovery of consciousness, and adverse event incidence.

## 2 Materials and methods

This systematic review follows the PRISMA 2020 guideline (Page et al., 2021). Preferred Reporting Items for Systematic Reviews and Meta-Analyses (PRISMA) statement was followed in our study. This meta-analysis was prospectively registered in INPLASY (202470131).

### 2.1 Inclusion criteria

Type of study (S): Randomised controlled trial (RCTs); Type of participant (P): Patients undergoing general anesthesia surgery, regardless of race, age, gender, type of surgery; Intervention (I): SMI or SFI administered preoperatively, intraoperatively, or perioperatively (specific timing varies by study; see Table 1), while undergoing conventional surgical treatment.

**TABLE 1:** Characteristics and intervention details of the included studies.

Study (author, year)	Number (T/C)	Age (T/C)	Gender (male/female)		Preoperative MMSE score (mean + SD)		Type of surgery	Interventions		Manufacturing company	Dosage	Course of treatment	Diagnostic criteria for POCD	Drop-outs	Adverse reactions	Informed consent	Ethical review	Target outcomes	Fund	Country
			T	C	T	C		T	C											
Sun et al. (2023)	40/40	66.19 ± 5.37/65.83 ± 5.28	23/17	21/19	28.21 ± 1.05	28.30 ± 1.09	Radical resection of pulmonary carcinoma	SMI 50 mL + 250 mL GS	300 mL GS	Shenwei pharmaceutical group Co., Ltd	50 mL	Once, intraoperative + Qd, 7 days after surgery	MMSE score <27	Not	Not	Yes	NM	1/2	NM	China
Zhou et al. (2023)	35/35	55.40 ± 7.86/58.37 ± 7.20	25/10	21/14	27.33 ± 1.06	27.68 ± 1.17	Thoracic surgery	SMI 60 mL + 250 mL GS	Blank control	Dali pharmaceutical Co., Ltd	60 mL	Once, intraoperative	MMSE score <27	Not	NM	Yes	NM	1/2	Yes	China
Chen (2019)	45/45	74.23 ± 2.85/73.12 ± 3.66	25/20	23/22	29.13 ± 0.91	28.98 ± 1.01	NM	SMI 60 mL + 250 mL GS	310 mL GS	Yunnan Gejiu Biopharmaceutical Co., Ltd	60 mL	Once, intraoperative	Decrease in MMSE score greater than 1 standard deviation	Not	NM	NM	NM	1/2	Yes	China
Chu et al., 2016	30/30	NM	NM	NM	28.1 ± 1.1	28.5 ± 0.8	Radical resection of esophageal cancer	SMI 0.6 mL/kg + 250 mL NS	NS	Ya’an Sanjiu pharmaceutical Co., Ltd	0.6 mL/kg	Once, intraoperative	MMSE score <26	Not	NM	NM	NM	1/2	NM	China
Xu and Wang (2016)	43/43	68.5 ± 3.7/69.3 ± 3.5	27/16	28/15	28.92 ± 1.1	28.78 ± 1.2	Knee replacement	SMI 50 mL + 150 mL NS	200 mL NS	Dali pharmaceutical Co., Ltd	50 mL	Once, intraoperative	MMSE score ≤23	Not	NM	Yes	NM	1/2	NM	China
Qiao (2016)	48/47	67.6 ± 4.8/66.9 ± 5.0	25/23	27/20	28.8 ± 1.1	29.3 ± 0.9	Lumbar surgery	SMI 50 mL + 250 mL GS	300 mL GS	Hebei Shenwei pharmaceutical Co., Ltd	50 mL	Once, intraoperative	MMSE score decreased by 2 points compared to preoperative	Not	Not	Yes	Yes	1/2	NM	China
Wang et al. (2015)	40/40	71.8 ± 5.5/72.3 ± 5.2	30/10	28/12	26.7 ± 1.5	26.4 ± 1.3	Knee replacement	SMI 50 mL + 150 mL NS	200 mL NS	Dali pharmaceutical Co., Ltd	50 mL	Once, intraoperative	MMSE score decreased by 2 points compared to preoperative	Not	NM	Yes	NM	1/2/4	Yes	China
Miao et al. (2014)	20/20	56 ± 10/53 ± 10	15/5	16/4	28.29 ± 1.20	28.15 ± 0.99	Cardiac valve replacement	SMI 0.6 mL/kg + 250 mL NS	Equal volume of NS	Ya’an Sanjiu pharmaceutical Co., Ltd	0.6 mL/kg	Once, intraoperative	Decrease in MMSE score greater than 1 standard deviation	Not	NM	Yes	Yes	1/2	Yes	China
Fang et al. (2014)	48/48	65–80/66–82	27/21	29/19	29.1 ± 0.9	28.7 ± 1.1	Hip replacement	SMI 60 mL + 250 mL GS	310 mL GS	Zhengda Qingchun Bao pharmaceutical Co., Ltd	60 mL	Once, intraoperative	Decrease in MMSE score greater than 1 standard deviation	Not	NM	Yes	Yes	1/2	NM	China
Yang et al. (2019)	80/80	68 ± 5/68 ± 4	NM	NM	26.52 ± 1.87	26.17 ± 2.62	Gynecological tumor surgery	SFI 100 mL + 250 mL GS	350 mL GS	NM	100 mL	Once, intraoperative	Decrease in MMSE score greater than 1 standard deviation	Not	NM	NM	NM	1/2/3	Yes	China
Dai et al. (2018)	28/28	42.4 ± 9.4/41.8 ± 8.6	14/14	12/16	27.2 ± 2.8	26.8 ± 3.1	Burn surgery	SFI 40 mL + NS	NS	Ya’an Sanjiu pharmaceutical Co., Ltd	40 mL	Once, intraoperative	MMSE score decreased by 2 points compared to preoperative	Not	Not	Yes	Yes	1/2	Yes	China
Tang et al. (2013)	45/45	69 ± 5/69 ± 4	28/17	26/19	26.62 ± 1.97	26.07 ± 2.52	Abdominal operation	SFI 100 mL + 250 mL GS	350 mL GS	NM	100 mL	Once, intraoperative	Decrease in MMSE score greater than 1 standard deviation	Not	NM	Yes	NM	1/2/3	Yes	China
Deng et al. (2013)	15/15	47.25 ± 11.47/50.52 ± 10.73	9/6	7/8	27.38 ± 1.24	27.42 ± 1.55	Aortic valve replacement	SFI 1.5 mL/kg + 250 mL GS	Equal volume of NS	Ya’an Sanjiu pharmaceutical Co., Ltd	1.5 mL/kg	Qd, 5 days before surgery + once, intraoperative	NM	Not	NM	Yes	Yes	1/3	Yes	China
Yuan et al. (2011a)	50/50	71 ± 4.8/70 ± 5.1	NM	NM	26.8 ± 1.4	26.5 ± 1.2	Orthopedic surgery	SFI 50 mL + 150 mL NS	200 mL NS	Ya’an Sanjiu pharmaceutical Co., Ltd	50 mL	Once, intraoperative	Decrease in MMSE score greater than 1 standard deviation	Not	NM	Yes	Yes	1/2/4	Yes	China
Yuan et al. (2011b)	25/25	40–69/40–69	NM	NM	27.5 ± 1.6	27.1 ± 1.3	Meningiomas operation	SFI 50 mL + 150 mL NS	200 mL NS	Ya’an Sanjiu pharmaceutical Co., Ltd	50 mL	Once, intraoperative	Decrease in MMSE score greater than 1 standard deviation	Not	Not	NM	NM	1/2/4	Yes	China
Zou et al. (2009)	8/8	39.0 ± 13.9/41.2 ± 11.3	4/4	3/5	28.24 ± 1.67	27.42 ± 1.55	Cardiac valve replacement	SFI (1 mL/kg)	Equal volume of NS	Ya’an Sanjiu pharmaceutical Co., Ltd	1 mL/kg	Once, intraoperative	MMSE score decreased by 1 point compared to preoperative	Not	NM	NM	NM	1/2/3	Yes	China

T, treatment group; C, control group; GS, glucose; MMSE, Mini-mental State Examination; NM, not mentioned; NS, normal saline; SFI, shenfu injection; SMI, shenmai injection; 1. Mini-mental State Examination; 2. Postoperative cognitive dysfunction; 3. Serum S100β Protein concentration; 4. Postoperative consciousness recovery time. This table reports only preoperative MMSE, values; postoperative MMSE, values at different time points are presented in forest plots (Figures 3, 4).

Control (C): Normal saline (NS) or Glucose (GS) or blank control while receiving conventional surgical treatment.

Outcome measures (O): MMSE score and incidence of POCD were selected as the primary outcomes, and secondary outcomes included serum S100β protein concentration, postoperative time to recovery of consciousness.

### 2.2 Exclusion criteria

1. Patients with preoperative cognitive impairment;
2. Studies that did not use MMSE scores for cognitive scoring;
3. Types of articles: Basic research literature such as reviews, comments, empirical studies, and mechanisms; Clinical research literature on non-randomized controlled trials; Literature on intervention measures other than SMI and SFI; Literature with incorrect or incomplete data; Duplicate literature.

### 2.3 Search strategy

Based on the PICO elements defined in Section 2.1 (Population: patients undergoing general anesthesia surgery; Intervention: Shenmai injection or Shenfu injection; Control: normal saline, glucose, or blank control; Outcomes: MMSE score, POCD incidence, serum S100β protein concentration, time to recovery of consciousness, and adverse events), we extracted keywords and descriptors for each element and developed search strings using Boolean operators (AND, OR). This study adheres to the GA best practice guidelines and the ConPhyMP reporting tool (Heinrich et al., 2022). Both SMI and SFI contain Red Ginseng (Panax ginseng C.A. Mey.) as a common botanical drug and are classified as Type A extracts according to the Chinese Pharmacopoeia. All commercial preparations included in the trials mostly adopt official national drug standards (National drug standard WS3-B-3428-98 for Shenmai Injection, 1998; National drug standard WS3-B-3427-98 for Shenfu Injection, 1998). However, the two injections differ in composition (SMI also contains Ophiopogon japonicus; SFI contains Aconitum carmichaelii) and are not chemically or pharmacologically interchangeable.

We searched a total of eight databases, PubMed, Embase, Web of Science, The Cochrane Library, CNKI, Wanfang, VIP, and CBM, to collect RCTs of interventions for postoperative cognitive function applying SMI and SFI from the time of database creation to 31 May 2025. We used these eight databases because the interventions (SMI and SFI) are primarily developed and used in China, and a substantial number of relevant RCTs are published only in Chinese journals. Therefore, searching multiple Chinese databases (CNKI, Wanfang, VIP, CBM) in addition to international databases (PubMed, Embase, Web of Science, Cochrane Library) was necessary to minimize language and regional bias and to ensure a comprehensive search. Search terms included those related to SMI and SFI (“shenmai injection” or “shenfu injection”) and those related to postoperative cognitive function (“cognition” or “dementia”) (Supplementary Table 1). There were no restrictions on date, sex, age, race, or type of surgery. In addition, references to relevant original articles were thoroughly reviewed for further relevant studies.

### 2.4 Data extraction

Two authors (QQL and LNC) independently screened the literature based on inclusion and exclusion criteria, while data were extracted and cross-checked according to a pre-designed form. To resolve disagreements by consensus, we used group consensus and consulted a third-party reviewer (WJP). Extracts: First author’s name, year of publication, sample size, age and sex of participants, type of surgery, intervention (including full name of preparation, manufacturer, dosage, and course of treatment), control intervention, outcome measures (MMSE scores, POCD incidence, S100β, consciousness recovery time, adverse events), and any reported information on preparation quality (e.g., batch number, chemical characterization, pharmacopoeial compliance).

### 2.5 Outcomes assessment

The primary efficacy outcome was the MMSE score from 1 h postoperatively to 7 days postoperatively. Secondary efficacy outcomes included the incidence of POCD from 3 to 7 days postoperatively and the incidence of postoperative adverse events. According to the 2018 nomenclature recommendations, perioperative neurocognitive disorders include cognitive decline diagnosed before operation, postoperative delirium, cognitive decline diagnosed up to 30 days after the procedure (delayed neurocognitive recovery, dNCR) and up to 12 months (postoperative neurocognitive disorder). The above diagnoses were made according to the Diagnostic and Statistical Manual of Mental Disorders, Fifth Edition (DSM-5). The diagnosis of POCD was based on cognitive decline in most of our included studies, so we continue to use the term “POCD” rather than “dNCR” in this article.

### 2.6 Quality evaluation

The methodological quality of the included studies was assessed using the Cochrane RoB 2.0 tool. This tool evaluates five domains of bias: (1) arising from the randomization process, (2) due to deviations from intended interventions, (3) due to missing outcome data, (4) in measurement of the outcome, and (5) in selection of the reported result, culminating in an overall risk-of-bias judgment. Two independent assessors (YCY and JFM) performed the RoB 2.0 assessments. Following independent evaluation, the assessors cross-checked their findings. Any discrepancies were resolved through consultation with a third reviewer (WJP).

### 2.7 Statistical analysis

Statistical processing was performed using Review Manager 5.4 software. Continuous variables were analysed using mean differences (MDs) or standardised mean differences (SMDs) and 95% confidence intervals (CIs). Relative risks (RRs) and 95% CIs were used for dichotomous data. Heterogeneity was analysed using the Q test and I^2^ statistics. If P > 0.10 or I^2^ < 50%, a fixed-effects model was used; otherwise, a random-effects model was selected. Sensitivity analyses and subgroup analyses were performed to assess the stability of the results and to detect potential sources of heterogeneity. Publication bias was assessed using funnel plots. Differences were considered statistically significant when P < 0.05.

### 2.8 Assessment of evidence quality

The GRADE approach was used to assess the quality of evidence for the outcomes, with two evaluators (JFM and YHP) conducting independent analyses to determine the quality of the evidence supporting each outcome. Any disagreements were resolved by the other evaluator (WJP).

## 3 Results

### 3.1 Literature search and screening

**FIGURE 1:**
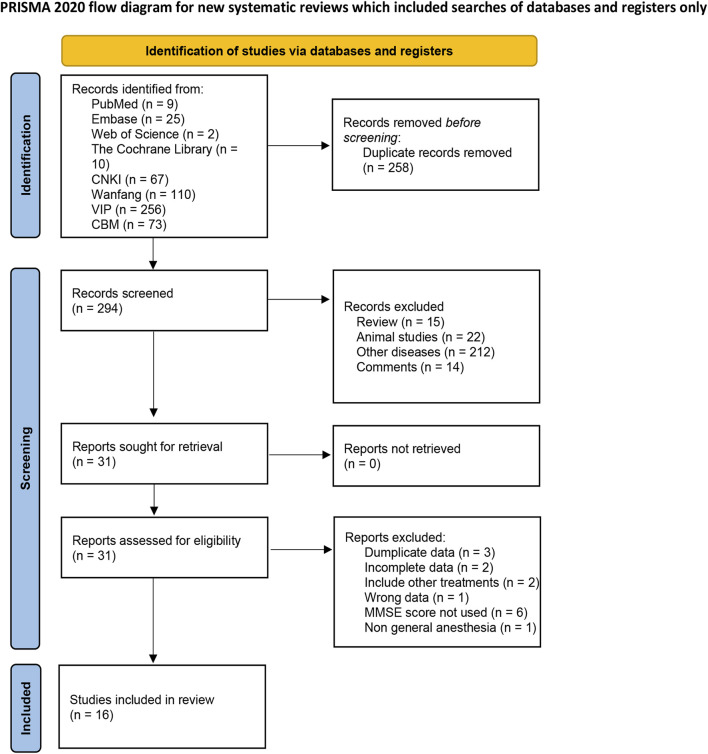
Flow diagram of literature screening.

Initially 552 relevant articles were found in eight databases and then 258 duplicates were checked by Endnote software. A total of 31 articles were obtained by reading the titles and abstracts of the articles. After reading the full text further, six of them were excluded because they did not use the MMSE scores, three were duplicates of reported data, and six were excluded because they lacked complete data, contained incorrect data, non-general anesthesia surgery, and contained other treatments. Finally, 16 Chinese articles were included (Figure 1).

### 3.2 Characteristics of studies

A total of 16 RCTs, involving 1,199 patients (600 in the intervention group and 599 in the control group), were included. Nine studies investigated SMI (Zhou et al., 2023; Sun et al., 2023; Chen, 2019; Chu and Sun, 2016; Xu and Wang, 2016; Qiao, 2016; Wang and Li, 2015; Miao et al., 2014; Fang et al., 2014) and seven examined SFI (Yang et al., 2019; Dai et al., 2018; Tang et al., 2013; Deng et al., 2013; Yuan et al., 2011a; Yuan et al., 2011b; Zou et al., 2009). Fifteen studies specified clear diagnostic criteria. One study did not report mean patient age, while four failed to report gender distribution. One trial compared SMI against no medication; the remaining studies used either normal saline or glucose as the control. However, the control types varied across studies: most used normal saline, some SMI studies used glucose solution, and Zhou et al. (2023) used a blank control (without specifying whether any intravenous fluid was administered). All included studies confirmed baseline comparability between the intervention and control groups. Detailed characteristics of the included studies are summarized in Table 1. To address the comparability of ginseng preparations across studies, we have added Supplementary Tables 2, 3, which systematically provide for each included study the following information as required by the ConPhyMP guidelines: full name of the preparation, botanical drug species and plant part used, preparation and extraction method, manufacturer, regulatory approval information, pharmacopoeial or quality standard reference, reported chemical profile, marker metabolites, whether batch-specific analytical information was reported, and whether sufficient information was provided to assess preparation quality and reproducibility. It must be explicitly stated that none of the 16 included studies reported batch-specific chemical data (e.g., quantitative ginsenoside content or chromatographic fingerprints), although all products comply with national pharmacopoeial standards. A complete ConPhyMP compliance checklist covering all reporting criteria is provided in Supplementary Table 4.

### 3.3 Quality evaluation of the included studies

**FIGURE 2:**
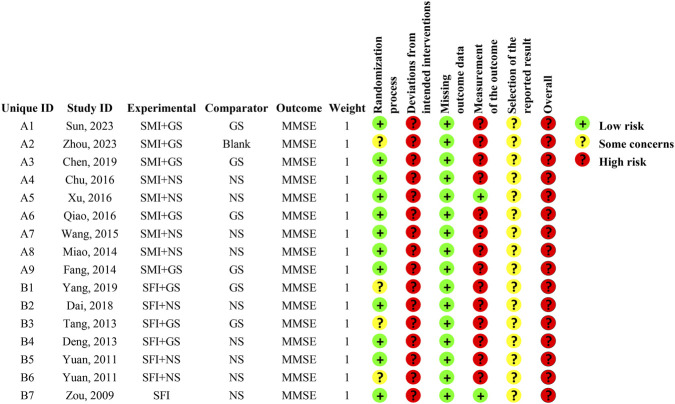
Risk of bias summary. Notes: RoB 2.0 domains: D1, randomization process; D2, deviations from intended interventions; D3, missing outcome data; D4, measurement of the outcome; D5, selection of the reported result. “+” indicates low risk of bias; “?” indicates some concerns; “−“ indicates high risk of bias.

The risk of bias assessment for included studies is summarized in Figure 2. Among the 16 studies analyzed, 12 specified methods for generating random sequences, while 4 merely mentioned “randomization” without detailing sequence generation. No study provided sufficient information on allocation concealment implementation, leaving uncertainty about its proper execution. Blinding of investigators/personnel was universally unreported, and only two studies mentioned blinding of outcome assessors. All studies maintained complete outcome data. Concerns regarding potential selective reporting bias resulted in an overall “some concerns” rating. All 16 included studies were judged to carry a high risk of bias due to these methodological limitations.

### 3.4 Meta-analysis results

#### 3.4.1 Postoperative MMSE scores

**FIGURE 3:**
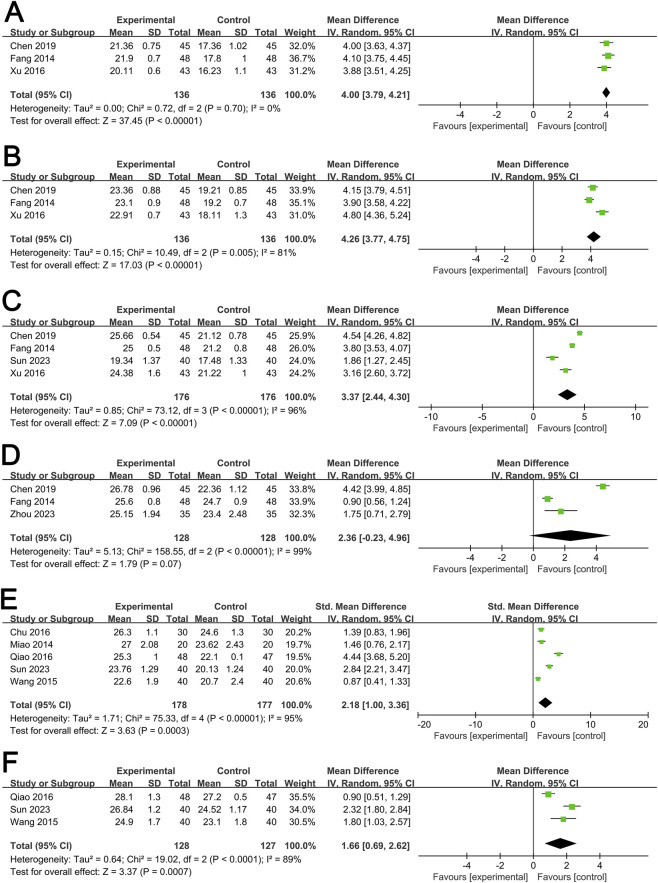
Forest plot of SMI improving postoperative MMSE. **(A)** 1 h after surgery; **(B)** 12 h after surgery; **(C)** 24 h after surgery; **(D)** 48 h after surgery; **(E)** 3 days after surgery; **(F)** 7 days after surgery. Notes: SMI, Shenmai injection; MD, mean difference; SMD, standardized mean difference; RR, relative risk; CI, confidence interval.

This analysis pooled data from 16 randomized controlled trials (1199 participants: SMI = 657, SFI = 542) evaluating MMSE scores for SMI and SFI separately compared to controls using MD. Analysis of three studies at 1-h and 12-h post-op showed significantly higher MMSE scores in the SMI group compared to controls (1 h: MD = 4.00, 95% CI 3.79 to 4.21, P < 0.001; Figure 3A; 12 h: MD = 4.26, 95% CI: 3.77 to 4.75, P < 0.001; Figure 3B). Four studies at 24 h also showed significantly higher SMI scores (MD = 3.37, 95% CI: 2.44 to 4.30, P < 0.001; Figure 3C). Using random-effects models due to significant heterogeneity, SMI did not show statistically significant scores at 48 h (3 studies; MD = 2.36, 95% CI: −0.23 to 4.96, P = 0.07; I^2^ = 99%; Figure 3D). Due to the extremely high heterogeneity (I^2^ = 99%), this pooled result is presented only for exploratory purposes; the true effect range may include no effect or even a negative effect (prediction interval: −7.82–12.54). Significantly higher scores at 3 days (5 studies; MD = 2.18, 95% CI: 1.00 to 3.36, P < 0.001; I^2^ = 87%; Figure 3E), given the high heterogeneity (I^2^ = 87%), these findings should be interpreted with caution. There were also significantly higher scores at 7 days (3 studies; MD = 1.66, 95% CI: 0.69 to 2.62, P < 0.001; I^2^ = 76%; Figure 3F). Given the substantial heterogeneity (I^2^ = 76%), these findings should be interpreted with caution.

**FIGURE 4:**
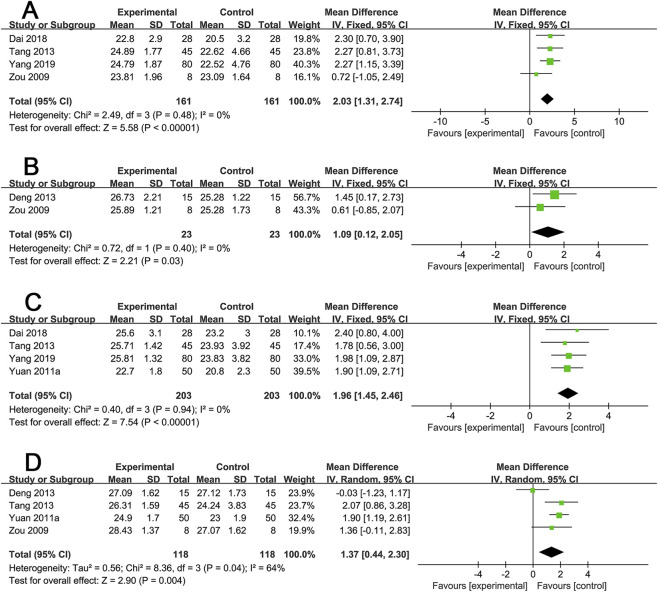
Forest plot of SFI improving postoperative MMSE. **(A)** 24 h after surgery; **(B)** 2 days after surgery; **(C)** 3 days after surgery; **(D)** 7 days after surgery. Notes: SFI, Shenfu injection; MD, mean difference; SMD, standardized mean difference; RR, relative risk; CI, confidence interval.

Four studies reported significantly higher MMSE scores in the SFI group versus control at 24 hours postoperatively (MD = 2.03, 95% CI: 1.31 to 2.74, P < 0.001; Figure 4A), with no significant heterogeneity (P = 0.48, I^2^ = 0%). At 2 days (2 studies), fixed-effects analysis showed higher SFI scores (MD = 1.09, 95% CI: 0.12 to 2.05, P < 0.01; Figure 4B). Four studies at 3 days showed significantly higher SFI scores (MD = 1.96, 95% CI: 1.45 to 2.46, P < 0.001; Figure 4C). For 7-day outcomes (4 studies), random-effects analysis (significant heterogeneity: P = 0.04, I^2^ = 64%) indicated higher MMSE scores in the SFI group (MD = 1.37, 95% CI: 0.44 to 2.30, P < 0.01; Figure 4D). Due to moderate heterogeneity (I^2^ = 64%), these results should be interpreted with caution.

#### 3.4.2 Outcomes of the incidence of POCD within 3 days and at day 7 postoperatively

**FIGURE 5:**
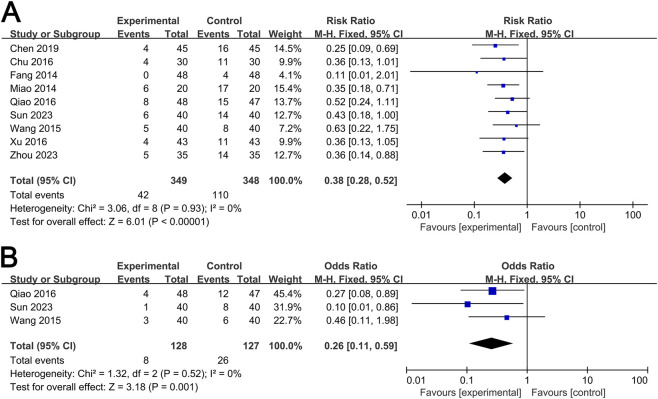
Forest plot of SMI reducing POCD incidence. **(A)** POCD incidence within 3 days postoperatively; **(B)** POCD incidence at 7 days postoperatively. Notes: SMI, Shenmai injection; MD, mean difference; SMD, standardized mean difference; RR, relative risk; CI, confidence interval.

This analysis pooled data from 1,169 participants (SMI: 657; SFI: 512). Nine studies reported POCD incidence within 3 days postoperatively, involving 349 SMI participants versus 348 controls. Fixed-effects meta-analysis (no significant heterogeneity: P = 0.88, I^2^ = 0%) showed significantly lower POCD incidence in the SMI group (RR = 0.38, 95% CI: 0.28 to 0.52, P < 0.001; Figure 5A). Three studies assessing POCD incidence at 7 days also showed significantly reduced rates in the SMI group (RR = 0.26, 95% CI: 0.11 to 0.59, P < 0.05; Figure 5B).

**FIGURE 6:**
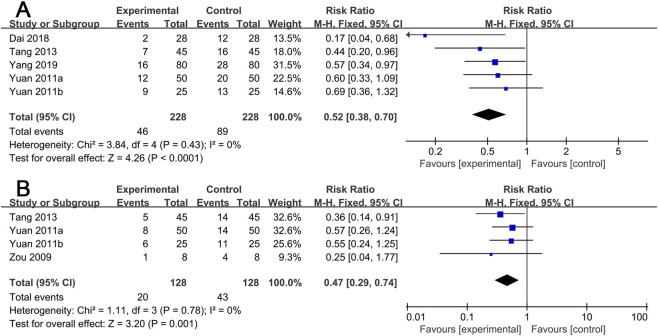
Forest plot of SFI reducing POCD incidence. **(A)** POCD incidence within 3 days postoperatively; **(B)** POCD incidence at 7 days postoperatively. Notes: SFI, Shenfu injection; MD, mean difference; SMD, standardized mean difference; RR, relative risk; CI, confidence interval.

Five studies evaluated POCD incidence within 3 days postoperatively in the SFI group (228 participants) versus control (228 participants). Fixed-effects meta-analysis (no significant heterogeneity: P = 0.43, I^2^ = 0%) showed significantly lower POCD incidence in the SFI group (RR = 0.52, 95% CI: 0.38 to 0.70, P < 0.001; Figure 6A). Four studies assessing POCD at 7 days also showed significantly reduced incidence in the SFI group (RR = 0.47, 95% CI: 0.29 to 0.74, P < 0.01; Figure 6B).

#### 3.4.3 Serum S100β protein concentration after surgery

**FIGURE 7:**
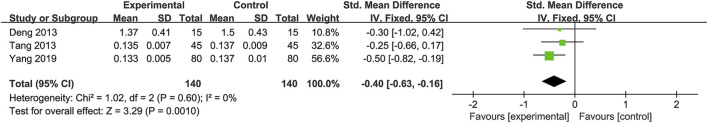
Forest plot of postoperative S100β.Notes: SMI, Shenmai injection; SFI, Shenfu injection; MD, mean difference; SMD, standardized mean difference; RR, relative risk; CI, confidence interval.

Four RCTs (Yang et al., 2019; Tang et al., 2013; Deng et al., 2013; Zou et al., 2009) evaluated perioperative serum S100β protein concentration changes, all of which used SFI. Analysis of three studies (SFI: N = 140, Control: N = 140) at 24 h postoperatively showed no significant heterogeneity (P = 0.60, I^2^ = 0%). Fixed-effects meta-analysis showed significantly lower S100β concentrations in the SFI group versus control (SMD = −0.40, 95% CI: −0.63 to −0.16, P < 0.05; Figure 7). Additionally, one trial (Tang et al., 2013) showed that serum S100β peaked at surgery completion, with significantly lower concentrations in the SFI group at 1 h postoperatively compared to controls.

#### 3.4.4 Postoperative recovery time of consciousness

**FIGURE 8:**
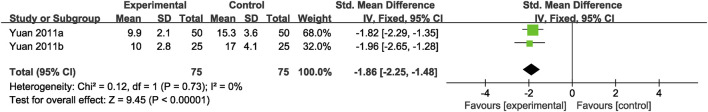
Forest plot of consciousness recovery time of SFI. Notes: SFI, Shenfu injection; MD, mean difference; SMD, standardized mean difference; RR, relative risk; CI, confidence interval.

Three RCTs (Wang and Li, 2015; Yuan et al., 2011a; Yuan et al., 2011b) involving 40 SMI (vs. 40 control) and 75 SFI (vs. 75 control) participants assessed time to postoperative consciousness recovery. For the SFI group, two RCTs (Yuan et al., 2011a; Yuan et al., 2011b) showed significantly shorter postoperative recovery time (MD = −1.86 min, 95% CI: −2.25 to −1.48, P < 0.001; Figure 8). For the SMI group, one RCT (Wang and Li, 2015) also showed a significantly shorter recovery time (MD = −5.40 min, P < 0.01).

#### 3.4.5 Subgroup analysis

##### 3.4.5.1 Dose subgroup analyses

**FIGURE 9:**
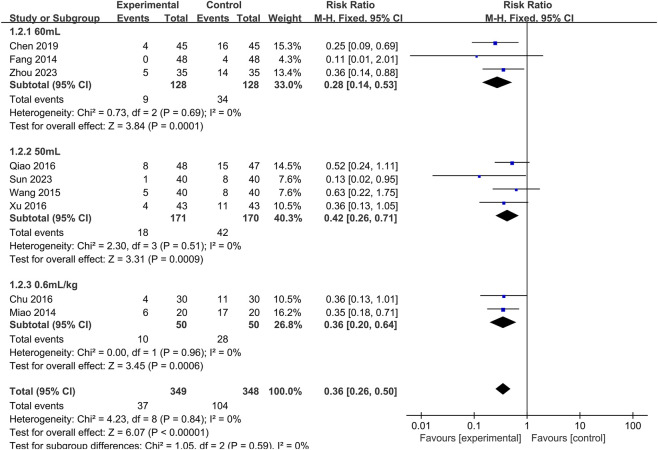
Subgroup analysis of SMI doses. Notes: SMI, Shenmai injection; RR, risk ratio; CI, confidence interval.

**FIGURE 10:**
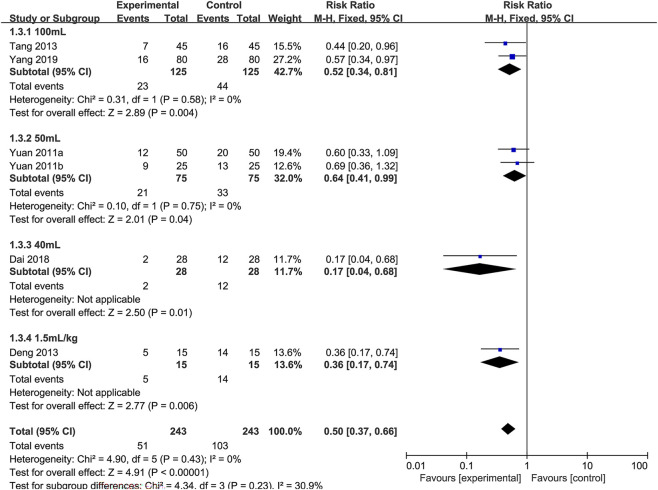
Subgroup analysis of SFI doses. Notes: SFI, Shenfu injection; RR, risk ratio; CI, confidence interval.

Given identical administration frequencies, subgroup analysis based on SMI dose (Figures 9, 10) showed significantly reduced POCD incidence across all doses: 60 mL (RR = 0.28, 95% CI: 0.14 to 0.53, P < 0.001), 50 mL (RR = 0.42, 95% CI: 0.26 to 0.71, P < 0.001), and 0.6 mL/kg (RR = 0.36, 95% CI: 0.20 to 0.64, P < 0.001).

Subgroup analysis of SFI doses showed significantly reduced POCD incidence across all regimens: 100 mL (RR = 0.52, 95% CI: 0.34 to 0.81, P < 0.05), 50 mL (RR = 0.64, 95% CI: 0.41 to 0.99, P < 0.05), 40 mL (RR = 0.17, 95% CI: 0.04 to 0.68, P < 0.05), and 1.5 mL/kg (RR = 0.36, 95% CI: 0.17 to 0.74, P < 0.05).

A trend toward reduced POCD incidence was observed across all dose subgroups. However, due to the small number of studies per subgroup, the lack of dose continuity, and the incomparability between weight-based and fixed-volume dosing, the current evidence is insufficient to determine an optimal dose.

##### 3.4.5.2 Subgroup analysis of POCD incidence by diagnostic criteria

**FIGURE 11:**
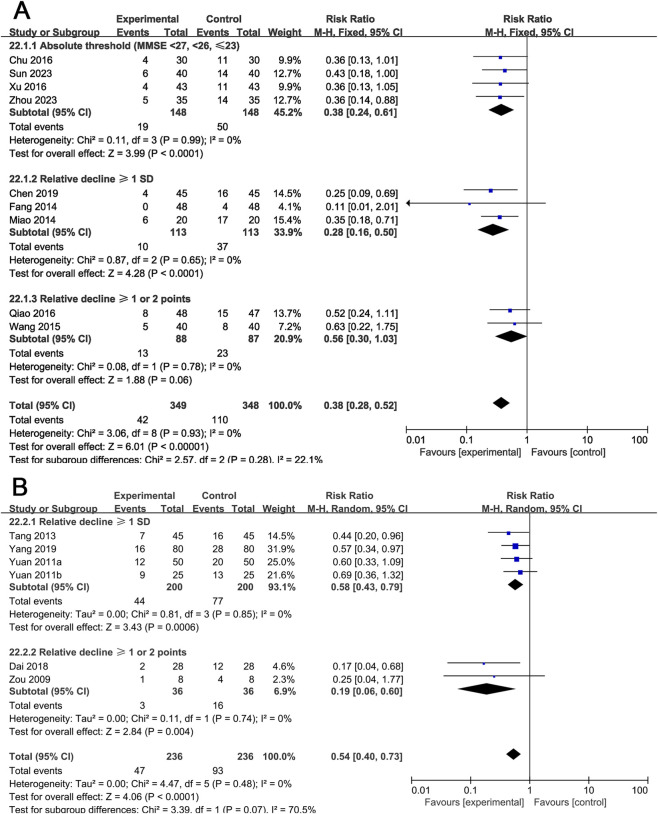
Subgroup analysis of POCD incidence by diagnostic criteria. **(A)** Shenmai injection (SMI); **(B)** Shenfu injection (SFI). Notes: RR, risk ratio; CI, confidence interval.

To address the inconsistency in POCD diagnostic criteria across studies, we performed subgroup analyses stratified by diagnostic criteria (Figure 11). The 16 studies were categorized into three groups: absolute threshold (MMSE <27, <26, ≤23; 4 studies), relative decline ≥1 standard deviation (7 studies), and relative decline ≥1 or two points (4 studies).

For SMI (Figure 11A), the pooled RR for the absolute threshold group was 0.38 (95% CI: 0.24 to 0.61; I^2^ = 0%); for the relative decline ≥1 SD group, 0.28 (95% CI: 0.16 to 0.50; I^2^ = 0%); and for the relative decline ≥1 or two points group, the individual study RRs were 0.52 (Qiao et al., 2016) and 0.63 (Wang et al., 2015), with a pooled RR of 0.56 (95% CI: 0.30 to 1.03; I^2^ = 0%). The direction of effect was consistently protective (RR < 1) across all three subgroups, and the subgroup difference was not statistically significant (P = 0.28).

For SFI (Figure 11B), the pooled RR for the relative decline ≥1 SD group (4 studies) was 0.58 (95% CI: 0.43 to 0.79; I^2^ = 0%); for the relative decline ≥1 or two points group (2 studies), the pooled RR was 0.19 (95% CI: 0.06 to 0.60; I^2^ = 0%). Again, both subgroups showed a protective direction, and the subgroup difference was not statistically significant (P = 0.07).

These findings indicate that despite heterogeneity in diagnostic criteria, the direction of association of SMI and SFI on POCD incidence remains consistent across different definitions, with no reversal of effect.

#### 3.4.6 Adverse reactions

Among the 16 included RCTs, only three studies reported adverse events. One trial reported one case of nausea each in the SMI intervention and control groups (Sun et al., 2023). Another trial documented adverse events during SFI administration (Dai et al., 2018): one case of allergy, three of dizziness, and six of chest tightness in the SFI group, versus zero allergies, four dizziness cases, and eight chest tightness cases in controls. No adverse events (including allergic reactions, dizziness, gastrointestinal effects, or respiratory depression) were observed in two RCTs (Qiao, 2016; Yuan et al., 2011b). The remaining 12 trials did not report adverse event information. It is important to note that the majority of included trials did not systematically report adverse events, and therefore no reliable safety assessment can be derived from the RCT evidence alone.

To provide a more comprehensive safety profile, we supplemented our analysis with large-scale post-marketing surveillance data. For SMI, a national analysis covering 4,220 spontaneous reports and 32,358 hospital-monitored cases showed an Adverse Drug Reaction (ADR) incidence of about 0.093%, with main events being chest tightness, chills, pruritus, palpitations, fever, and nausea; serious ADRs were rare (Wang et al., 2015). For SFI, a prospective, multicenter, registry-based study (30,106 patients, 28 hospitals, 2012–2015) identified 23 confirmed ADRs, giving an incidence of 0.076% (95% CI: 0.045%–0.108%). Main ADRs included rash, pruritus, injection site discomfort, nausea, vomiting, abdominal pain, dizziness, chest tightness, palpitations, chills, fever, and dyspnea. No serious ADRs were detected (Wang et al., 2017). However, these post-marketing data cannot compensate for the inadequate adverse event reporting in the included RCTs and should not be interpreted as evidence of safety.

#### 3.4.7 Publication bias analysis and sensitivity analysis

**FIGURE 12:**
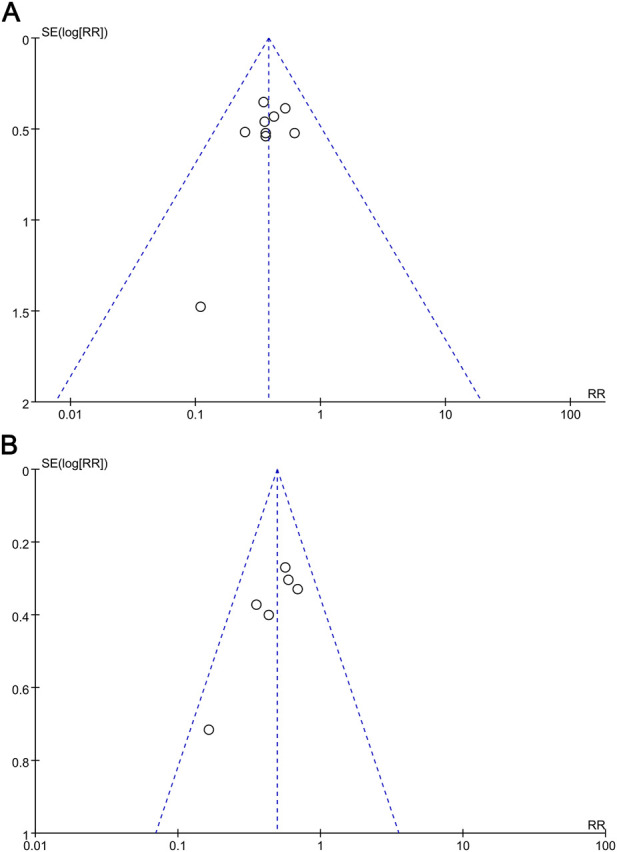
Filled funnel plot for the publication bias. Notes: RR, risk ratio; SE, standard error.

Sensitivity analysis was performed by sequentially removing each included study to assess result stability. For the SMI group, removing one trial (Xu and Wang, 2016)—which used a distinct drug dose causing high heterogeneity—significantly reduced heterogeneity in 12-h MMSE scores. In the SFI group, excluding one trial (Deng et al., 2013)—noted for its highest administration frequency—markedly reduced heterogeneity in 7-day MMSE scores. No substantial changes in effect sizes or significance occurred upon excluding any other studies, indicating robust results. In addition, to address control group variability, we performed a sensitivity analysis by excluding studies that used glucose solution as the control in the SMI group (Sun et al., 2023; Qiao et al., 2016; Fang et al., 2014; Chen et al., 2019) or blank control (Zhou et al., 2023), retaining only those that used normal saline as the control. The direction and magnitude of the pooled effects for POCD incidence remained consistent with the main analysis, with no statistically significant change in effect sizes, indicating that control type variability did not decisively influence the overall conclusions. Funnel plot analysis of 3-day POCD incidence in the SMI (Figure 12A) and SFI (Figure 12B) groups suggested potential asymmetry, which may indicate publication bias or other small-study effects. Moreover, all included studies were at high risk of bias, which itself could lead to overestimation of treatment effects. Readers should interpret our findings with extreme caution.

#### 3.4.8 Overall quality of evidence according to GRADE

Using the GRADE framework, the quality of evidence for each outcome was rated. Because all included studies were at high risk of bias (lack of allocation concealment and blinding), the evidence quality for POCD was low. The evidence for MMSE scores, S100β protein levels, and time to consciousness recovery was rated low. The primary reasons for downgrading were small sample sizes in the included RCTs and risk of bias. The quality ratings of the included evidence are detailed in Table 2. Consequently, the statistically significant pooled estimates reported in Sections 3.4.1–3.4.5 should not be interpreted as strong evidence of clinical efficacy. Given the low certainty of evidence, future high-quality research is likely to change these estimates.

**TABLE 2:** Quality of evidence.

Quality assessment							No of patients		Effect	Quality
Outcomes (no. Of studies)	Design	Risk of bias	Inconsistency	Indirectness	Imprecision	Other considerations	Intervention	Comparison	Absolute
MMSE (16)	RCT	Very serious^a^	Not serious	serious^b^	Not serious	None	600	599	​	⊕⊕Οο Low
POCD (16)	RCT	Very serious^a^	Not serious	Not serious	Not serious	None	600	599	​	⊕⊕Οο Low
S100β (4)	RCT	Very serious^a^	Not serious	Not serious	serious^c^	None	140	140	SMD = −0.40, 95% CI (−0.63, −0.16)	⊕⊕Οο Low
Postoperative consciousness recovery time (3)	RCT	Very serious^a^	Not serious	Not serious	serious^c^	None	115	115	MD = −5.67,95% CI (−6.46, −4.87)	⊕⊕Οο Low

^a^ the design of the experiment with a large bias in random, distributive hiding or blind.

^b^ heterogeneity test (I2 > 50%).

^c^ not meeting the optimal information size criteria, primarily due to small sample size.

## 4 Discussion

### 4.1 Summary of the results

To our knowledge, this is the first meta-analysis evaluating the efficacy and safety of SMI and SFI for improving postoperative cognitive function. Pooling data from 16 RCTs (1,199 patients), the analysis showed that both SMI and SFI were associated with significantly higher postoperative MMSE scores, reduced POCD incidence, and shortened time to consciousness recovery compared to controls. Subgroup analyses by dose suggested a trend toward reduced POCD incidence across all dose subgroups, but the differences between subgroups were not statistically significant, and no firm conclusion regarding an optimal dose could be drawn due to limited sample sizes and methodological constraints. Treatment groups exhibited significantly lower postoperative serum S100β protein concentrations—a potential neurodamage biomarker inversely correlated with cognitive function—further supporting cognitive improvement (Barbu et al., 2022). Because none of the included SMI studies measured serum S100β, this meta-analysis cannot assess the effect of SMI on this biomarker. Safety analysis revealed no statistically significant difference in adverse event rates between treatment and control groups. Only a few studies reported adverse events, and the reported events were mostly mild. However, because the majority of studies did not report safety information, the current evidence does not allow a reliable conclusion regarding the overall safety of ginseng-containing injections. These results suggest that SMI and SFI, each prepared according to national pharmacopoeial standards, may each be associated with improved postoperative neurocognitive outcomes. However, the evidence is limited due to high heterogeneity and risk of bias. As detailed in the GRADE assessment (Section 3.4.8), all included trials were at high risk of bias, and the certainty of evidence is low; therefore, the statistically significant findings should be considered preliminary rather than definitive, and the two preparations should not be considered interchangeable.

Another important issue relates to the heterogeneity in diagnostic criteria and the limitations of the MMSE. The included studies used heterogeneous POCD diagnostic criteria (absolute MMSE thresholds, ≥1 SD decline, or a decline of 1-2 points), and although subgroup analyses showed a consistent direction of effect (Figures 11A,B), the lack of standardization limits comparability across studies and the precision of pooled estimates. More importantly, relying on the MMSE to define POCD has several important concerns. The MMSE is known to have ceiling effects and low sensitivity for detecting mild neurocognitive changes (Spencer et al., 2013; FrancoMarina et al., 2010), which may lead to underestimation of true POCD incidence and failure to capture subtle postoperative cognitive declines. Furthermore, the clinical meaningfulness of statistically significant MMSE differences is uncertain, as a 1-2 point increase, especially when scores are already in the normal range, may not translate into clinically noticeable benefit. Therefore, conclusions based on MMSE-defined POCD must be interpreted cautiously, and future studies should adopt more sensitive tools such as the Montreal Cognitive Assessment (MoCA) or comprehensive neuropsychological batteries, along with standardized diagnostic criteria (Trzepacz et al., 2015; Ciesielska et al., 2016).

Growing recognition of POCD has heightened focus on its management, with comprehensive perioperative strategies expected to synergize with interventions to reduce POCD risk (Safavynia et al., 2022; Kong et al., 2022). While POCD mechanisms remain unelucidated, animal and human studies implicate postoperative neuroinflammation and oxidative stress as key pathogenic factors (Barreto Chang et al., 2022; Liu et al., 2023; Yang et al., 2020). Li et al. showed elevated hippocampal reactive oxygen species (ROS) and pro-inflammatory cytokines in aged rats after hip fracture surgery; intracerebroventricular Nrf2 activator injection upregulated hippocampal Nrf2 expression, activating antioxidant defenses, reducing ROS/cytokines, and ameliorating POCD (Li et al., 2023). A prospective observational study revealed significantly increased serum CHI3L1 and IL-6 levels in POCD patients 1 week post-hip arthroplasty versus non-POCD controls, suggesting inflammatory involvement in pathogenesis (Zheng et al., 2023). Furthermore, Panax ginseng or its extracts show potential therapeutic benefits as cognitive adjuvant therapy, potentially via neuroinflammation reduction (Malik and Tlustos, 2023; Lorca et al., 2023). A large prospective cohort study indicates regular intake of Panax ginseng as a botanical drug may lower cognitive impairment risk, with neuroprotective effects potentially mediated by attenuated inflammatory responses and oxidative damage (Tao et al., 2023). However, non-randomized studies, whether animal, *in vitro*, or human observational, only suggest biological plausibility, not clinical evidence.

The TCM theoretical basis of SMI and SFI is summarized in Supplementary Table 5, which may help international readers understand the rationale for these botanical drug injections. SMI and SFI are traditional Chinese medicine injections developed per TCM principles. SMI (prepared from Panax ginseng and Ophiopogon japonicus, clinically used since the 1970s) tonifies qi, alleviates deficiency, and nourishes bodily fluids. SFI (Panax ginseng and Aconitum carmichaelii extract, approved 1987) reinforces qi, counters fatigue, and revives yang qi. Perioperative qi imbalance and blood deficiency (“qi deficiency state”) align with TCM pathology addressed by both ginseng-based formulas. Processed Panax ginseng (steamed/dried) contains bioactive ginsenosides (Rb1, Rb2, Rc, Rd, Re, Rg1) and secondary metabolites (Rh2, Rg3) formed during deglycosylation/decarboxylation (Ye et al., 2023; Chopra et al., 2023). Preclinical studies demonstrate perioperative SMI/SFI administration accelerates consciousness recovery, lowers S100β/IL-6, and prevents POCD in aged rats—potentially via anti-inflammatory/antioxidant mechanisms (Zhang et al., 2018). Ginsenoside Rg1 mitigates anesthesia/surgery-induced cognitive impairment (Miao et al.), while ginsenosides improve isoproterenol-anesthesia-impaired memory functions (Xu et al., 2022). Bioinformatic analysis suggests PI3K/Akt-mediated neuroinflammation modulation underpins SMI’s anti-POCD effects (Yi et al., 2024).

Recent studies suggest gut microbiota may critically regulate neuroinflammation (Mao et al., 2021). Lian et al. linked POCD to adverse alterations in gut microbiota and fecal metabolites, observing decreased relative abundance of unclassified *Bacteroides*, Mucispirillum, and unclassified Clostridia, alongside increased Actinobacteria and Escherichia-Shigella post-anesthesia/surgery compared to baseline controls (Lian et al., 2021). A prospective study demonstrated transcutaneous electrical acupoint stimulation (TEAS) reduced cumulative POCD duration in laparoscopic cancer surgery patients, potentially via modulation of gut-brain axis-associated inflammatory factors (Liu et al., 2021). Both red ginseng and ginseng reportedly regulate gut microbiota, potentially reducing oxidative stress and neuroinflammation by increasing probiotics and suppressing inflammatory bacteria (Peng et al., 2021). Li et al. found Korean red ginseng administration increased *Lactobacillus* abundance, restored blood-brain barrier integrity, reduced microglial activation, and improved cognition in mice (Lee et al., 2022). A systematic review of 26 studies concluded microbiota-targeted therapies effectively prevent POCD and enhance cognitive function (Sugita et al., 2023).

Some biochemical studies on ginsenosides may be susceptible to pan-assay interference (PAINS), a phenomenon where certain metabolites produce false-positive results in various assays (Baell and Holloway, 2010). This concern applies to mechanistic extrapolations from *in vitro* or animal studies, but not to the clinical conclusions of this systematic review, which are based solely on RCTs. The outcomes we analyzed (MMSE scores, POCD incidence, S100β levels, and consciousness recovery time) are not derived from PAINS-prone assays. Therefore, our main claims regarding the efficacy and safety of SMI and SFI are not compromised by PAINS concerns.

### 4.2 Significance and limitations of the study

This PRISMA-guided meta-analysis evaluated the efficacy and safety of SMI and SFI for preventing POCD. Subgroup analyses by dose suggested a trend toward reduced POCD incidence across different dose levels, but due to the small number of studies per subgroup, the lack of dose continuity, and the incomparability between weight-based and fixed-volume dosing, the current evidence is insufficient to determine an optimal dose. Any suggestion of a dose-related effect should be considered hypothesis-generating. As products integrating traditional knowledge with contemporary pharmaceutical production, SMI and SFI offer rapid onset and practical administration (Huajuan et al., 2023). These injections thus represent a botanical drug-based complementary approach to postoperative neuroprotection.

This analysis has several constraints. First and most importantly, the included studies lacked sufficient information on the chemical comparability of SMI and SFI preparations. Although all products complied with national pharmacopoeial standards (Type A extracts), none of the original RCTs reported batch-specific data such as ginsenoside content, fingerprints, or marker metabolite quantification. This omission has several consequences. First, it is unknown whether the preparations used across different trials were chemically consistent, which may have contributed to the observed clinical heterogeneity. Second, the lack of chemical characterization prevents assessment of reproducibility and limits the generalizability of our findings. Third, because SMI and SFI have different botanical compositions (SMI contains Ophiopogon japonicus; SFI contains Aconitum carmichaelii), they should not be considered interchangeable. Our separate analyses of SMI and SFI are exploratory and do not imply equivalence. Future studies must report full chemical characterization according to ConPhyMP guidelines to enable meaningful comparisons and meta-analyses.

Secondly, literature searches were limited to English and Chinese publications, introducing potential language bias and possibly compromising review comprehensiveness. Korean, Japanese, and other language databases were not searched, which may have missed potentially relevant studies not published in English or Chinese.

Thirdly, all included studies were at high risk of bias (no allocation concealment, no blinding of personnel, and only two studies reported blinding of outcome assessors). Consequently, the GRADE evidence quality for all outcomes was downgraded to low, and the findings may overestimate the true treatment effects.

Fourthly, only three of the 16 included RCTs reported adverse events, and most studies did not systematically collect or report safety data during follow-up. We acknowledge that the original RCTs under-reported safety data; however, external post-marketing surveillance data cannot compensate for this deficiency and do not establish safety within the current RCT evidence base. Therefore, safety remains insufficiently established. Regarding drug interactions, based on national drug standards and official package inserts, SMI should not be mixed with other drugs in the same container, nor with antibiotics; it is also incompatible with Veratrum and Polygala per TCM theory (Ministry of Health, 1998). SFI should not be used with Pinellia, Trichosanthes, Fritillaria, Ampelopsis, Bletilla, or Veratrum; it should also avoid direct mixing with aminophylline, coenzyme A, vitamin K, or catecholamines (Ministry of Health, 1998). Additionally, some of the included studies were published relatively early (e.g., 2009 and 2011), and their methodological design and reporting quality may not meet current standards, which could introduce some bias.

Fifthly, the control interventions varied across studies: Glucose solution, as an active control, may have metabolic effects that could theoretically influence cerebral energy metabolism and cognitive function, potentially diluting or enhancing the observed treatment effect. Although a sensitivity analysis excluding non-saline controls did not materially alter the main findings, this variability remains a limitation that should be considered when interpreting the results. In addition to dose, we explored potential sources of heterogeneity such as surgery type, patient age, and baseline cognitive status. Due to the limited number of included studies, formal meta-regression was not feasible; however, qualitative observations suggest that these factors may contribute to the observed heterogeneity.

Finally, heterogeneity in POCD diagnostic criteria across studies may affect the precise quantitative interpretation of the pooled effect. While subgroup analyses showed a consistently protective direction across all diagnostic criteria (P for subgroup differences = 0.28 for SMI and 0.07 for SFI), the exact effect size may vary depending on the threshold used. Additionally, publication bias may be present (most studies were small and reported positive results), and the lack of allocation concealment and blinding in most studies may introduce detection and selection bias. Therefore, the conclusions of this meta-analysis should be considered exploratory rather than definitive. Nevertheless, the consistent direction and magnitude of effects across multiple studies provides preliminary but meaningful evidence to inform clinical decision-making. Although methodological flaws may have influenced the effect estimates to some extent, the high consistency across studies suggests that the potential clinical value of SMI and SFI for improving postoperative cognitive outcomes warrants further investigation.

## 5 Conclusion

This systematic review and meta-analysis evaluated the efficacy and safety of SMI and SFI separately for the prevention of POCD. Low-certainty evidence suggests that each preparation may be associated with improved MMSE scores and reduced POCD incidence compared to controls. However, because SMI and SFI have different chemical compositions and pharmacological profiles, they are not interchangeable, and our separate analyses should be interpreted as exploratory. The current evidence does not permit comparative conclusions between the two preparations. Furthermore, due to high risk of bias, heterogeneity, and the lack of batch-specific chemical characterization in the original studies, definitive claims of efficacy or safety cannot be made. Moreover, as all included studies defined POCD using the MMSE, a tool with limited sensitivity for subtle cognitive changes, the clinical meaningfulness of the observed improvements is uncertain and the findings should be interpreted with caution. High-quality RCTs with adequate blinding, standardized diagnostic criteria, and full reporting of preparation chemistry are urgently needed.

From a clinical perspective, these findings suggest that SMI and SFI could be considered as an adjunctive option for postoperative cognitive protection, particularly in high-risk patients (e.g., elderly individuals or those with pre-existing cognitive vulnerability) or in settings where conventional preventive measures have limited effect. Clinical decisions should be individualized, weighing potential benefits against the low-to-moderate quality of evidence. If used, close monitoring for adverse events is advised, and patients should be encouraged to participate in well-designed prospective RCTs to strengthen the evidence base.

While showing promise for POCD prevention, these findings require validation through high-quality, large-sample, multicenter, prospective, double-blinded RCTs. Future research should: 1) Deepen exploration of botanical drug injections for postoperative cognitive improvement to establish reference treatment paradigms; 2) Implement rigorously designed RCTs with standardized diagnostic criteria, extended follow-up, and comparative dose/regimen studies to inform pharmacological management; 3) Enhance safety profiling to clarify contraindications and compatibility risks, guiding standardized clinical application; 4) Incorporate more sensitive cognitive assessment tools (e.g., MoCA) and establish POCD-specific minimal clinically important difference (MCID) thresholds to facilitate clinically meaningful interpretation of results.

## Data Availability

The original contributions presented in the study are included in the article/Supplementary Material, further inquiries can be directed to the corresponding authors.
