# Supplementary material for: Efficacy and safety of Shenmai injection and Shenfu injection on postoperative cognitive dysfunction: a systematic review and meta-analysis
Source: Front Pharmacol. 2026 Jun 30;17:1664949. doi: 10.3389/fphar.2026.1664949 (PMC13364905; doi:10.3389/fphar.2026.1664949)
Supplement: Supplementary file 1 [file Supplementaryfile1.docx]

Supplementary Material

**Supplementary Table 1. Search strategy to identify RCTs in databases.**

| **Databases** | **Search strategy** |
| --- | --- |
| PubMed | ((("Dementia"[Mesh]) OR "Cognition"[Mesh] OR "Dementia*" OR "Cogniti*")) AND ((shenmai injection) OR (shenfu injection)) |
| Embase | #1 'shenmai injection'/exp OR 'shenmai injection'  #2 'shenfu injection'/exp OR 'shenfu injection'  #3 'cognition'/exp OR 'cognition'  #4 'dementia'/exp OR 'dementia'  #5 #1 OR #2  #6 #3 OR #4  #7 #5 AND #6 |
| Web of science | #1 TS = (cognition) OR TS = (dementia)  #2 TS = (shenmai injection) OR TS = (shenfu injection)  #3 #1 AND #2 |
| The Cochrane library | #1 MeSH descriptor: [Dementia]  #2 MeSH descriptor: [Cognition]  #3 (Dementia*): ti,ab,kw  #4 (Cogniti*): ti,ab,kw  #5 (shenmai injection): ti,ab,kw  #6 (shenfu injection): ti,ab,kw  #7 #1 OR #2 OR #3 OR #4  #8 #5 OR #6  #9 #7 AND #8 |
| CNKI | ((SU = '参麦注射液') OR (SU = '参附注射液')) AND ((SU = '认知') OR (SU = '痴呆')) |
| Wanfang | 主题: (("参麦注射液" OR "参附注射液") AND ("认知" OR "痴呆")) |
| VIP | (U = (参麦注射液 OR 参附注射液)) AND (U = (认知 OR 痴呆)) |
| CBM | ("认知" [常用字段:智能] OR "痴呆" [常用字段:智能]) AND ("参麦注射液" [常用字段:智能] OR "参附注射液" [常用字段:智能] ) |

**Supplementary Table 2. The details of SMI** **in the included study.**

| **Study (Author, Year)** | **Name** | **Source** | **Composition** | **Drug Standard Reference** | **Drug Approval Number** | **Preparation and extraction method** | **Quality Analysis and Standard Compliance** | **Taxonomic verification source** | **Traditional Chinese Medicine actions** |
| --- | --- | --- | --- | --- | --- | --- | --- | --- | --- |
| Sun et al., 2023 | Shenmai  injection | Shenwei Pharmaceutical Group Co., Ltd | Panax ginseng C.A.Mey. [Araliaceae; Radix Ginseng Rubra]  Ophiopogon japonicus (Thunb.) Ker Gawl. [Asparagaceae; Ophiopogonis radix] | National Drug Standard of the China Food and Drug Administration, WS3-B-3428-98-2010Z | Z13020886 | https://www.dayi.org.cn/drug/1147846.html. | In accordance with national drug standard; qualitative and quantitative analyses of the marker metabolites ginsenosides Rg1, Re, and Rb1 were carried out (manufacturer data). No batch-specific analytical results reported in the original trial. | <https://powo.science.kew.org/taxon/urn:lsid:ipni.org:names:91472-1.>  <https://powo.science.kew.org/taxon/urn:lsid:ipni.org:names:429781-1.> | Replenishing Qi to arrest desertion, nourishing Yin to generate fluids, and restoring the pulse. |
| Zhou et al., 2023 | Shenmai  injection | Dali Pharmaceutical Co., Ltd | Panax ginseng C.A.Mey. [Araliaceae; Radix Ginseng Rubra]  Ophiopogon japonicus (Thunb.) Ker Gawl. [Asparagaceae; Ophiopogonis radix] | National Drug Standard of the China Food and Drug Administration, WS3-B-3428-98-2010Z | Z20093647 |  |  |  |  |
| Chen et al., 2019 | Shenmai  injection | Yunnan Gejiu Biopharmaceutical Co., Ltd | Panax ginseng C.A.Mey. [Araliaceae; Radix Ginseng Rubra]  Ophiopogon japonicus (Thunb.) Ker Gawl. [Asparagaceae; Ophiopogonis radix] | National Drug Standard of the China Food and Drug Administration, WS3-B-3428-98-2010Z | Z53021720 |  |  |  |  |
| Chu et al., 2016 | Shenmai  injection | Ya'an Sanjiu Pharmaceutical Co., Ltd | Panax ginseng C.A.Mey. [Araliaceae; Radix Ginseng Rubra]  Ophiopogon japonicus (Thunb.) Ker Gawl. [Asparagaceae; Ophiopogonis radix] | National Drug Standard of the China Food and Drug Administration, WS3-B-3428-98-2010Z | Z51021845 |  |  |  |  |
| Xu et al., 2016 | Shenmai  injection | Dali Pharmaceutical Co., Ltd | Panax ginseng C.A.Mey. [Araliaceae; Radix Ginseng Rubra]  Ophiopogon japonicus (Thunb.) Ker Gawl. [Asparagaceae; Ophiopogonis radix] | National Drug Standard of the China Food and Drug Administration, WS3-B-3428-98-2010Z | Z20093648 |  |  |  |  |
| Qiao et al., 2016 | Shenmai  injection | Hebei Shenwei Pharmaceutical Co., Ltd | Panax ginseng C.A.Mey. [Araliaceae; Radix Ginseng Rubra]  Ophiopogon japonicus (Thunb.) Ker Gawl. [Asparagaceae; Ophiopogonis radix] | National Drug Standard of the China Food and Drug Administration, WS3-B-3428-98-2010Z | Z13020887 |  |  |  |  |
| Wang et al., 2015 | Shenmai  injection | Dali Pharmaceutical Co., Ltd | Panax ginseng C.A.Mey. [Araliaceae; Radix Ginseng Rubra]  Ophiopogon japonicus (Thunb.) Ker Gawl. [Asparagaceae; Ophiopogonis radix] | National Drug Standard of the China Food and Drug Administration, WS3-B-3428-98-2010Z | Z20093648 |  |  |  |  |
| Miao et al., 2014 | Shenmai  injection | Ya'an Sanjiu Pharmaceutical Co., Ltd | Panax ginseng C.A.Mey. [Araliaceae; Radix Ginseng Rubra]  Ophiopogon japonicus (Thunb.) Ker Gawl. [Asparagaceae; Ophiopogonis radix] | National Drug Standard of the China Food and Drug Administration, WS3-B-3428-98-2010Z | Z51021845 |  |  |  |  |
| Fang et al., 2014 | Shenmai  injection | Zhengda Qingchun Bao Pharmaceutical Co., Ltd | Panax ginseng C.A.Mey. [Araliaceae; Radix Ginseng Rubra]  Ophiopogon japonicus (Thunb.) Ker Gawl. [Asparagaceae; Ophiopogonis radix] | National Drug Standard of the China Food and Drug Administration, WS3-B-3428-98-2010Z | Z33020021 |  |  |  |  |

**Supplementary Table 3. The details of SFI in the included study.**

| **Study (Author, Year)** | **Name** | **Source** | **Composition** | **Drug Standard Reference** | **Drug Approval Number/ Batch number** | **Preparation and extraction method** | **Quality Analysis and Standard Compliance** | **Aconitum alkaloids content** | **Taxonomic verification source** | **Traditional Chinese Medicine actions** |
| --- | --- | --- | --- | --- | --- | --- | --- | --- | --- | --- |
| Yang et al., 2019 | Shenfu injection | Not reported in the original study | Panax ginseng C.A.Mey. [Araliaceae; Radix Ginseng Rubra]  Aconitum carmichaelii Debeaux [Ranunculaceae; Aconiti Lateralis Radix Praeparata (Heishunpian) | Not reported in the original study | Not reported in the original study | https://www.dayi.org.cn/drug/1147516.html. | Not reported in the original study | Not reported in the original study | <https://powo.science.kew.org/taxon/urn:lsid:ipni.org:names:91472-1.>  https://powo.science.kew.org/taxon/urn:lsid:ipni.org:names:707241-1. | Restoring Yang to rescue the patient from collapse, and replenishing Qi to arrest desertion. |
| Dai  et al., 2018 | Shenfu injection | Ya'an Sanjiu Pharmaceutical Co., Ltd | Panax ginseng C.A.Mey. [Araliaceae; Radix Ginseng Rubra]  Aconitum carmichaelii Debeaux [Ranunculaceae; Aconiti Lateralis Radix Praeparata (Heishunpian) | National Drug Standard of the China Food and Drug Administration, WS3-B-3427-98-2013 | Z20043116 |  | In accordance with national drug standard; qualitative identification and quantitative determination of the marker metabolite ginsenoside Rb1 were carried out (manufacturer data). No batch-specific analytical results reported in the original trial. | Calculated as aconitine (C_3_₄H₄₇NO₁₁), not more than 0.1 mg per mL. |  |  |
| Tang et al., 2013 | Shenfu injection | Not reported in the original study | Panax ginseng C.A.Mey. [Araliaceae; Radix Ginseng Rubra]  Aconitum carmichaelii Debeaux [Ranunculaceae; Aconiti Lateralis Radix Praeparata (Heishunpian) | Not reported in the original study | Not reported in the original study |  | Not reported in the original study | Not reported in the original study |  |  |
| Deng et al., 2013 | Shenfu injection | Ya'an Sanjiu Pharmaceutical Co., Ltd | Panax ginseng C.A.Mey. [Araliaceae; Radix Ginseng Rubra]  Aconitum carmichaelii Debeaux [Ranunculaceae; Aconiti Lateralis Radix Praeparata (Heishunpian) | Ministerial Standard of the Ministry of Health for Chinese Patent Medicine, Volume 18, WS3-B-3427-98 | Batch number: 110109 |  | In accordance with national drug standard; qualitative identification and quantitative determination of the marker metabolite ginsenoside Rb1 were carried out (manufacturer data). No batch-specific analytical results reported in the original trial. | Calculated as aconitine (C_3_₄H₄₇NO₁₁), not more than 0.1 mg per mL. |  |  |
| Yuan^a^et al., 2011 | Shenfu injection | Ya'an Sanjiu Pharmaceutical Co., Ltd | Panax ginseng C.A.Mey. [Araliaceae; Radix Ginseng Rubra]  Aconitum carmichaelii Debeaux [Ranunculaceae; Aconiti Lateralis Radix Praeparata (Heishunpian) | Ministerial Standard of the Ministry of Health for Chinese Patent Medicine, Volume 18, WS3-B-3427-98 | Batch number: 090204 |  | In accordance with national drug standard; qualitative identification and quantitative determination of the marker metabolite ginsenoside Rb1 were carried out (manufacturer data). No batch-specific analytical results reported in the original trial. | Calculated as aconitine (C_3_₄H₄₇NO₁₁), not more than 0.1 mg per mL. |  |  |
| Yuan^b^ et al., 2011 | Shenfu injection | Ya'an Sanjiu Pharmaceutical Co., Ltd | Panax ginseng C.A.Mey. [Araliaceae; Radix Ginseng Rubra]  Aconitum carmichaelii Debeaux [Ranunculaceae; Aconiti Lateralis Radix Praeparata (Heishunpian) | Ministerial Standard of the Ministry of Health for Chinese Patent Medicine, Volume 18, WS3-B-3427-98 | Z51020664 |  | In accordance with national drug standard; qualitative identification and quantitative determination of the marker metabolite ginsenoside Rb1 were carried out (manufacturer data). No batch-specific analytical results reported in the original trial. | Calculated as aconitine (C_3_₄H₄₇NO₁₁), not more than 0.1 mg per mL. |  |  |
| Zou et al., 2009 | Shenfu injection | Ya'an Sanjiu Pharmaceutical Co., Ltd | Panax ginseng C.A.Mey. [Araliaceae; Radix Ginseng Rubra]  Aconitum carmichaelii Debeaux [Ranunculaceae; Aconiti Lateralis Radix Praeparata (Heishunpian) | Ministerial Standard of the Ministry of Health for Chinese Patent Medicine, Volume 18, WS3-B-3427-98 | Batch number: 071102 |  | In accordance with national drug standard; qualitative identification and quantitative determination of the marker metabolite ginsenoside Rb1 were carried out (manufacturer data). No batch-specific analytical results reported in the original trial. | Calculated as aconitine (C_3_₄H₄₇NO₁₁), not more than 0.1 mg per mL. |  |  |

**Supplementary Table 4. GA-online Best Practice Tool (ConPhyMP) – Completed Checklists (Tables 1 and 2A)**

| **Journal (target)** | Frontiers in Pharmacology |
| --- | --- |
| **Manuscript** | Efficacy and Safety of Ginseng-containing Injections on Postoperative Cognitive Dysfunction: A Systematic Review and Meta-Analysis |
| **Date** | 2026-5-25 |

**ConPhyMP Table 1. ConPhyMP checklist of information for reporting plant material and its initial processing (relevant for all studies on medicinal and food plants including extract types A, B, and C)**

| **Section/Topic** | **Item** | **Checklist item** | **Yes/No/Not applicable** | **Location in manuscript** |
| --- | --- | --- | --- | --- |
| Title and abstract | 1 | A clear and concise title including an informative abstract and balanced summary. | Yes | Title; Abstract |
| Description of the botanical drug and taxonomic authentication | 2 | Botanical or morphological authentication of the plant material (desirable is a combination with DNA barcoding, e.g., PCR, RFLP, genome sequencing) and the information must be included in a separate section of Material and Methods, if applicable, combined with the information required under item 3. | Yes (appraisal, based on pharmacopoeial botanical identification; no DNA barcoding reported) | Methods (Section 2.6 Quality Evaluation); Supplementary Tables 2-3 (taxonomic validity/identity fields, where reported) |
| Description of the extract and extraction process | 3 | A separate section in Material and Methods, covers the relevant information on the material investigated, including the full species name(s), authorites and family; e.g. Salvia miltorrhiza Bunge [Lamiaceae; Salviae miltorrhizae radix et rhizoma], and on the processing and extraction of the crude drug including the traditional processing of the material used medicinally (fumigation, steaming, roasting, cooking, frying, etc.). | Partially met (preparation method defined by pharmacopoeial standards but not detailed in original RCTs) | Supplementary Tables 2-3 (source/drug approval number/batch number, where reported) |
| Documentation of the legal basis for collection and processing | 4 | Full compliance with the Nagoya protocol, CITES, and all associated treaties including phytosanitary regulations. | Not applicable | Not applicable |
| Description of product characteristics, in case of a finished (commercial) product | 5 | Information on the characteristics of the commercial products including batch number and date of production/best by information and regulatory status. | Yes (where reported) | Table 2 (manufacturing company/dosage, where reported); Supplementary Tables 2-3 (source/drug approval number/batch number, where reported) |

**ConPhyMP Table 2A. ConPhyMP checklist of items for conducting and reporting analytical Methods relevant for extract type A (for species or botanical drugs covered in a monograph in one of the national or regional pharmacopoeias).**

| **Section/Topic** | **Item** | **Checklist item** | **Yes/No/Not applicable** | **Location in manuscript** |
| --- | --- | --- | --- | --- |
| Type of extract | 1 | A – Confirm that the species or botanical drug under investigation is covered in a monograph in one of the national or regional pharmacopoeias. | Yes (appraisal) | Supplementary Tables 2-3 (drug standard reference, where reported) |
| Preferred/main methods for extract characterisation/chemical analysis | 2 | Compliance with pharmacopoeial standards to be followed: (a) The description of the active ingredients in the botanical drug (if known) or analytical marker compounds as defined. (b) An analysis as defined in the monograph is needed if the extract has not been supplied with a certificate. (c) If the preparation was purchased, the manufacturer and certificate of analysis need to be included. Including either the preferred or alternative approaches for characterisation: (a) Triple chemical fingerprinting methods, each with one or more detection parameters. (b) Quantification of at least two marker compounds (unless this is not feasible, evidence needs to be provided), and justification of the choice of markers (if applicable). | No | None of the 16 included RCTs reported detailed chemical characterisation methods on marker compounds (e.g., ginsenoside fingerprints or quantitative data). Although all products are manufactured according to Chinese Pharmacopoeia standards (which define marker metabolites and require fingerprinting for quality control), this information was not provided in the original trial publications. Therefore, the included studies do not meet ConPhyMP Table 2A Item 2 expectations. This limitation is discussed in the main text (Discussion 4.5). |
| Alternative methods for extract characterisation/chemical analysis | 3 | (a) Single chemical fingerprinting method with at least three different detection parameters (i.e., altered detection parameters, like TLC/HPTLC with different staining reagents and/or UV excitation wavelengths, HPLC-DAD/LCDAD with different wavelengths). The same applies to coupling MS or NMR to chromatographic techniques. (b) Quantification of at least two marker compounds (unless this is not feasible, evidence needs to be provided), and justification of the choice of markers (if applicable). | No | Not reported in any of the included RCTs; no batch-specific fingerprints or quantitative marker metabolite data were provided. |
| Use of reference standards | 4 | (a) Direct overlay of the chromatogram of the sample with that of an officially specified reference standard (if applicable). (b) Chromatographic fingerprinting: Direct overlay of the chromatogram of the sample with that of official reference standards of the powdered plant material or the dry extract from the plant material. | No | Reference standard chromatograms not shown in original RCTs |
| Comparison of different extracts/samples of the same plants | 5 | (a) Direct comparison of the chromatographic/spectroscopic system and/or scoring system for “similarity” to be followed. | Not applicable |  |

**References**

Heinrich M, Jalil B, Abdel-Tawab M, Echeverria J, Kulić Ž, McGaw LJ, et al. (2022). Best Practice in the chemical characterisation of extracts used in pharmacological and toxicological research—The ConPhyMP—Guidelines. *Frontiers in Pharmacology.* 13:953205. doi:10.3389/fphar.2022.953205.

**Supplementary Table 5. Traditional Chinese Medicine theoretical basis of SMI and SFI.**

| **Aspect** | **Shenmai Injection (SMI)** | **Shenfu Injection (SFI)** |
| --- | --- | --- |
| Traditional Chinese Medicine pathogenesis | Perioperative Qi-Yin deficiency occurs as surgery and anesthesia deplete Qi and consume yin fluids, manifesting as fatigue, dry mouth, and lethargy. | Perioperative Yang collapse refers to severe Qi deficiency complicated by Yang exhaustion, presenting with cold limbs, a weak pulse, and delayed consciousness recovery. |
| Therapeutic principle | Replenishing Qi to arrest desertion, nourishing Yin to generate fluids, and restoring the pulse. | Restoring Yang to rescue the patient from collapse, and replenishing Qi to arrest desertion. |
| Composition (botanical drugs) | Panax ginseng C.A.Mey. [Araliaceae; Radix Ginseng Rubra]  Ophiopogon japonicus (Thunb.) Ker Gawl. [Asparagaceae; Ophiopogonis radix] | Panax ginseng C.A.Mey. [Araliaceae; Radix Ginseng Rubra]  Aconitum carmichaelii Debeaux [Ranunculaceae; Aconiti Lateralis Radix Praeparata (Heishunpian) |
| Traditional Chinese Medicine actions of each botanical drug component | Red ginseng: Greatly tonifies original Qi, generates fluids, and calms the spirit.  Ophiopogon: Nourishes Yin, moistens the lungs, and clears heart fire. | Red ginseng: Supplements Qi and arrests collapse.  Aconite (prepared): Restores Yang and rescues collapse, warms the meridians. |
| Synergy | Ginseng and Ophiopogon together reinforce Qi and Yin, preventing dehydration and cognitive decline after surgery. | Ginseng and Aconite together “rescue Yang from collapse”, rapidly reversing shock‑like states and promoting arousal. |
| Modern pharmacological correlates | Ginsenosides (Rg1, Re, Rb1) exert anti‑inflammatory, antioxidant, and anti‑apoptotic effects.  Ophiopogon saponins protect against ischemia/reperfusion injury. | Ginsenosides provide neuroprotection.  Aconite alkaloids (benzoylaconine, etc.) have cardiotonic, anti‑inflammatory, and stress‑modulating properties; they enhance cerebral microcirculation. |
